# ASIC-dependent LTP at multiple glutamatergic synapses in amygdala network is required for fear memory

**DOI:** 10.1038/srep10143

**Published:** 2015-05-19

**Authors:** Po-Han Chiang, Ta-Chun Chien, Chih-Cheng Chen, Yuchio Yanagawa, Cheng-Chang Lien

**Affiliations:** 1 Institute of Neuroscience, National Yang-Ming University, Taipei 112, Taiwan; 2 Institute of Brain Science, National Yang-Ming University, Taipei 112, Taiwan; 3 Molecular Medicine Program, Taiwan International Graduate Program, Institute of Biomedical Sciences, Academia Sinica, Taipei 115, Taiwan; 4 Institute of Biomedical Sciences, Academia Sinica, Taipei 115, Taiwan; 5 Department of Genetic and Behavioral Neuroscience, Gunma University Graduate School of Medicine and JST, CREST, Maebashi 371-8511, Japan; 6 Brain Research Center, National Yang-Ming University, Taipei 112, Taiwan

## Abstract

Genetic variants in the human ortholog of acid-sensing ion channel-1a subunit (ASIC1a) gene are associated with panic disorder and amygdala dysfunction. Both fear learning and activity-induced long-term potentiation (LTP) of cortico-basolateral amygdala (BLA) synapses are impaired in ASIC1a-null mice, suggesting a critical role of ASICs in fear memory formation. In this study, we found that ASICs were differentially expressed within the amygdala neuronal population, and the extent of LTP at various glutamatergic synapses correlated with the level of ASIC expression in postsynaptic neurons. Importantly, selective deletion of ASIC1a in GABAergic cells, including amygdala output neurons, eliminated LTP in these cells and reduced fear learning to the same extent as that found when ASIC1a was selectively abolished in BLA glutamatergic neurons. Thus, fear learning requires ASIC-dependent LTP at multiple amygdala synapses, including both cortico-BLA input synapses and intra-amygdala synapses on output neurons.

The amygdala is a complex structure in the mid-temporal lobe that plays a key role in fear learning and emotional processing^1^,^2^,^3^. Genetic variation in *ACCN2*, the human ortholog of the acid-sensing ion channel-1a subunit (*ASIC1a*) gene, is associated with both panic disorder and alterations in human amygdala structure and function^4^. Several lines of evidence implicate acid-sensing ion channels (ASICs) in the rodent amygdala in fear-like behavior. First, ASIC1a-null mice display impaired conditioned fear behavior and reduced innate fear^5^,^6^. Second, restoring ASIC1a in the basolateral complex of the amygdala (BLA) of ASIC1a-null mice rescues the fear memory impairments^7^. Third, overexpression of ASIC1a increases acquired fear-related behavior^8^. Finally, ASICs contribute to long-term potentiation (LTP) at cortico-BLA synapses, and this synaptic plasticity is critical for associative fear learning and memory^9^.

As proton-activated Na^+^-permeable channels, ASICs belong to the degenerin/epithelial Na^+^ channel superfamily^10^,^11^,^12^. To date, seven ASIC subunits (ASIC1a, 1b, 2a, 2b, 3, 4, and 5; a and b refer to splice variants), encoded by five genes (*ASIC1* to *ASIC5*), have been identified^13^,^14^. At least ASIC1a, ASIC2a, and ASIC2b are expressed in the central nervous system^11^,^15^,^16^,^17^. Of these, ASIC1a is the obligatory subunit of the ASIC^5^,^18^. In the brain, ASICs consist of homotrimeric ASIC1a and heterotrimeric ASIC1a/2a and ASIC1a/2b channels^19^,^20^. Although ASICs are expressed widely in the brain, ASIC1a and ASIC2 are particularly enriched in the amygdala^9^,^20^. The amygdala comprises a heterogeneous collection of nuclei, including the BLA, the central amygdala (CeA), and intercalated cell masses (ICMs)^1^,^3^,^21^. Fear conditioning is known to cause widespread synaptic plasticity in the amygdala^22^,^23^,^24^,^25^. Despite the importance of ASICs in the cortico-BLA for fear learning, it is not clear whether the distribution of ASICs varies among different cell types within amygdala circuits, or whether synaptic plasticity in other cell types depends on ASICs and such plasticity also plays a role in fear learning.

To address these questions, we measured ASIC currents in different types of amygdala neurons in acutely-prepared brain slices from young adult mice. We found that ASIC expression was highly cell type- and region-specific, whereas ASIC gating kinetics and subunit composition were similar. Notably, ASICs were highly expressed in glutamatergic principal neurons (PNs) of the BLA receiving cortical and thalamic inputs, and low-threshold bursting (LTB) gamma-aminobutyric acid (GABA)-ergic neurons in the medial sector of the CeA (CeM), which is the output station of the amygdala circuitry. Analysis of synaptic plasticity revealed that the abundance of ASICs in postsynaptic neurons correlated with, and contributed to, LTP of glutamatergic synapses and fear-like behavior. Furthermore, ASIC-dependent LTP at both cortico-BLA and intra-amygdala glutamatergic synapses was required for the formation of fear memories.

## Results

### Cell type-specific expression of ASICs in the BLA

**Figure 1:**
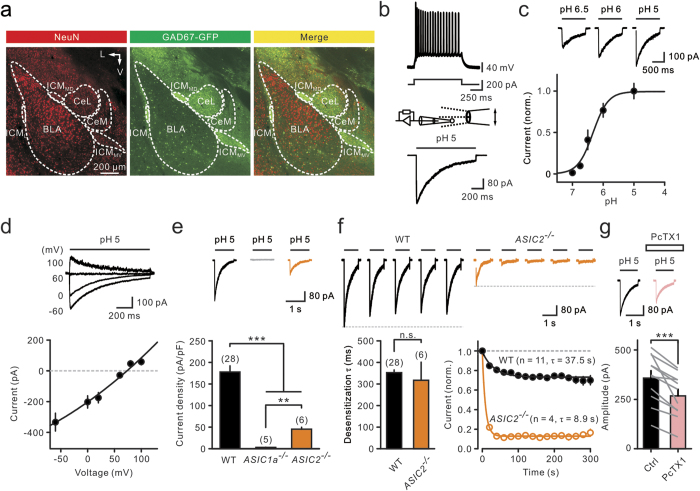
ASIC currents in mouse amygdala neurons were mediated by ASIC1a and ASIC2 subunits. (**a**) A coronal brain section from a *GAD67-GFP* KI mouse. Left, immunostaining of NeuN. Middle, GFP-expressing cells in the same slice. Right, merged image. BLA, basolateral amygdala; CeL/CeM, lateral/medial subdivisions of the central amygdala; ICM_MD_/ICM_MV_/ICM_L_, medial–dorsal/medial–ventral/lateral intercalated cell mass. Axis: L, lateral; V, ventral. (**b**) Top, firing pattern of a BLA-PN and the current protocol. Bottom, schematic of fast application of H^+^ to a nucleated patch and averaged ASIC current (from seven trials) recorded from the same cell. (**c**) Top traces, ASIC currents recorded from BLA-PNs. Bottom, ASIC current (normalized) plotted against pH value. The single Hill equation (half-maximal pH 6.3, Hill coefficient 1.8) was fitted to the data points. Points represent mean values from 4 to 20 experiments. (**d**) Top traces, ASIC currents recorded from a BLA-PN at different membrane potentials. Bottom, the current–voltage curve of ASIC currents. Data points are fitted with a polynomial function. (**e**) Top, example traces of ASIC currents recorded from BLA-PNs of WT, *ASIC1a*^–/–^ and *ASIC2*^–/–^ mice. Bottom, bar graph of the average current density. ^**^P < 0.01; ^***^P < 0.001, Wilcoxon rank-sum test. (**f**) Top, examples of ASIC currents recorded from BLA-PNs of WT and *ASIC2*^–/–^ mice; currents were evoked by repetitive 1 s pulses (from pH 7.4 to 5) at 0.05 Hz. Only the first five traces are shown. Bottom, bar graph of the average desensitization *τ* of ASIC currents (left) and time course of cumulative reduction during repeated stimulation (right). Current amplitudes were normalized to the first amplitude and were plotted against the time of each pulse. Continuous lines represent fits to a single exponential function; time constants for WT and *ASIC2*^–/–^ are 37.5 s and 8.9 s, respectively. (**g**) Top, ASIC currents recorded from BLA-PNs in the control and in the presence of 30 nM PcTX1. Traces are average of 3–7 sweeps. Bottom, PcTX1 significantly reduced ASIC currents. ^***^P < 0.001, Wilcoxon signed-rank test. Data obtained from the same cell are connected by solid lines.

The amygdala is composed of several synaptically interconnected nuclei^2^,^3^,^26^. The borders of individual nuclei in a coronal section of the mouse brain were outlined, and neurons were identified using the neuronal marker NeuN (Fig. 1a, left). To differentiate between GABAergic and non-GABAergic neurons, we used knock-in (KI) mice expressing the green fluorescent protein (GFP) in glutamate decarboxylase 67 (GAD67)-positive neurons^27^ (Fig. 1a, middle). Consistent with previous studies^2^,^21^,^26^,^27^,^28^, we found that the BLA consisted of a great majority (approximately 80%) of glutamatergic PNs and a minority of sparsely GABAergic interneurons (INs), whereas the CeA and ICMs were composed of mostly (approximately 90%) GFP-expressing cells (Fig. 1a, right).

To investigate the functional expression of ASICs, we used fast application of proton (H^+^) to excised nucleated patches from recorded neurons (Fig. 1b). As previously described^19^,^29^, this method allows rapid application of proton, an almost ideal space-clamp, and a reliable measurement of current density. Solution exchange could be achieved in less than 200 μs (see Methods) with our fast application system. We found that BLA-PNs generated regular and accommodating action potential (AP) trains, in response to depolarizing current pulses in the current clamp (Fig. 1b, top). Nucleated patches from BLA-PNs exhibited ASIC-like, transient inward currents (Fig. 1b, bottom), in response to a submillisecond switch of extracellular pH from 7.4 to 5. The peak amplitude of the current depended on the pH value of the applied solution (Fig. 1c).

As expected for a non-voltage-gated Na^+^ channel^11^, we found the reversal potential of ASIC-like currents to be 67.8 ± 5.2 mV (n = 6; Fig. 1d), close to the Na^+^ equilibrium potential (61 mV) under the recording condition. The current densities measured were independent of animal ages (19 to 30 days, 152 ± 4.5 pA/pF, n = 14; 30 to 60 days; 139 ± 28 pA/pF, n = 10; 60 to 75 days, 131 ± 8 pA/pF, n = 3; P = 0.50, Kruskal–Wallis test) and thus were pooled together in this study. On average, the current density was 178 ± 14 pA/pF in wild-type (WT) mice (n = 28; Fig. 1e). Using *ASIC1a*^–/–^ and *ASIC2*^–/–^ mice, we found that a pH fall from 7.4 to 5 did not activate detectable currents in *ASIC1a*^–/–^ mice (3.0 ± 0.6 pA/pF, n = 5; Fig. 1e), while these currents were greatly reduced in *ASIC2*^–/–^ mice (*ASIC2*^–/–^, 45.4 ± 4.5 pA/pF, n = 6; ^***^P < 0.001, Wilcoxon rank-sum test; Fig. 1e), consistent with the obligatory role of the ASIC1a subunit for a functional ASIC^30^ and the auxiliary role of ASIC2^31^. Furthermore, we found that although the desensitization τ was unchanged (Fig. 1f, bottom left), the current amplitude in *ASIC2*^–/–^ mice showed a significantly accelerated reduction during repeated stimulation (Fig. 1f, bottom right), Finally, these currents were significantly reduced by Psalmotoxin 1 (PcTX1) (control, 357 ± 38 pA; PcTX1, 268 ± 34 pA, n = 11; ^***^P < 0.001, Wilcoxon signed-rank test; Fig. 1g), a specific peptide blocker of ASIC1a homomeric, and ASIC1a/2b heteromeric, channels^32^,^33^. Together, these results indicate that proton-activated currents in BLA-PNs were mediated by ASIC1a, ASIC2a, and ASIC2b subunits. Owing to the weak sensitivity to PcTX1, a large proportion of current was likely mediated by ASIC1a/2a heteromers.

**Figure 2:**
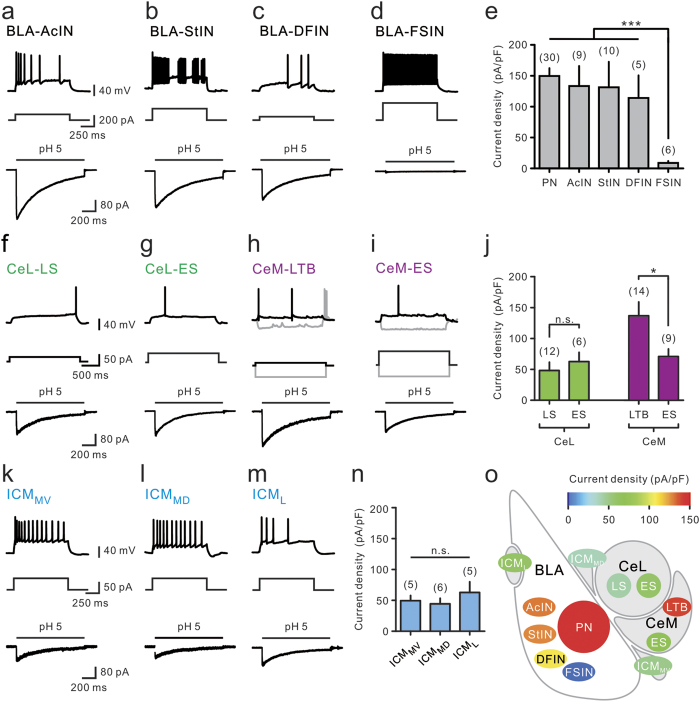
Mapping of ASIC expression in different types of amygdala neurons. (**a**–**d**) Top, examples of firing patterns recorded from BLA-INs as indicated and current protocols. Bottom, averaged ASIC currents recorded from the same cells (AcIN, from 13 trials; StIN, from 15 trials; DFIN, from 12 trials; FSIN, from 14 trials). Scale bars in (**a**) apply to (**b**) to (**d**). (**e**) Summ**a**ry of ASIC current **d**ensity of BLA neurons. (**f**–**i**) Top, examples of firing patterns recorded from neurons in the CeL and CeM. Bottom, averaged ASIC currents (LS, from 7 trials; ES, from 6 trials; CeM-LTB, from 9 trials; CeM-ES, from 14 trials) recorded from the same cell. Scale bars in (**f**) apply to (**g**) to (**i**). (**j**) Summary of ASIC current density. (**k**–**m**) Top, representative firing patterns of GABAergic neurons in the ICM_MV_, ICM_MD_, and ICM_L_. Bottom, ASIC currents recorded from the same cells. Scale bars in (**k**) apply to (**l**) and (**m**). (**n**) Summary of ASIC current density of ICM neurons. (**o**) Heat map summarizing ASIC current density of various cell types of amygdala neurons. Error bars represent mean±s.e.m. ^*^P < 0.05, ^***^P < 0.001; n.s., not significant, Wilcoxon rank-sum test.

GABAergic INs in the BLA display heterogeneous intrinsic excitability^34^,^35^. To avoid arbitrary and subjective classification, we performed hierarchical cluster analysis^36^ from 72 randomly recorded GFP-expressing cells in the BLA of *GAD67-GFP* KI mice. Four major IN subtypes were identified, including accommodating INs (AcINs), stuttering INs (StINs), delay-firing INs (DFINs), and fast-spiking INs (FSINs) (see Methods; Supplementary Table S1). Among these, AcINs exhibited an accommodating AP pattern (Fig. 2a; Supplementary Fig. S1); StINs exhibited a characteristic pattern of multiple AP bursts separated by variable quiescent periods (Fig. 2b; Supplementary Fig. S1); DFINs exhibited a marked spike delay in response to near-threshold current pulse injection (Fig. 2c; Supplementary Fig. S1); and FSINs showed characteristic brief APs with high firing rates and little accommodation (Fig. 2d; Supplementary Fig. S1). Nucleated patch measurements from these cells showed that AcINs, StINs, and DFINs exhibited large ASIC currents, similar to BLA-PNs, while FSINs showed very small ASIC currents (Fig. 2a, b, c, and d, bottom). Thus, ASIC expression in GABAergic INs in the BLA is cell type-specific (PN, 150 ± 13 pA/pF, n = 30; AcIN, 136 ± 32 pA/pF, n = 9; StIN, 131 ± 41 pA/pF, n = 10; DFIN, 114 ± 36 pA/pF, n = 5; FSIN, 8.9 ± 3.2 pA/pF, n = 6; Fig. 2e).

### Cell type- and subregion-specific ASIC expression in the CeA and ICMs

The CeA, which contains lateral (CeL) and medial (CeM) subdivisions, receives excitatory inputs from the BLA and inhibitory projections from the medial–dorsal ICM (ICM_MD_) and medial–ventral ICM (ICM_MV_)^3^. Previous studies have identified two major types (‘late-spiking’ [LS] and ‘early-spiking’ [ES]) of GABAergic neurons^37^,^38^. Consistently, our cluster analysis of CeL neurons revealed two non-overlapping cell populations (Supplementary Fig. S2; Supplementary Table S2), corresponding to LS and ES neurons, with a spike delay of 1769 ± 24 ms (n = 46) and 778 ± 86 ms (n = 26), respectively (P < 0.001, Wilcoxon rank-sum test). We found ASIC currents with modest and similar densities in LS and ES neurons (CeL-LS, 48 ± 13 pA/pF, n = 12; CeL*-*ES, 63 ± 15 pA/pF, n = 6; Fig. 2f, g, and j).

The CeM is the main output station of the amygdala circuitry^3^. We found that, in mouse amygdala, the CeM consisted of two major cell types, based on their firing properties: low-threshold bursting (LTB) cells (87/147, 59%; Fig. 2h) and ES cells (50/147, 34%; Fig. 2i; Supplementary Table S3). Unlike ES cells, LTB cells exhibited characteristic rebound APs after hyperpolarizing pulses (Fig. 2h) and showed significantly larger ASIC current densities than those found in ES cells (CeM-LTB, 143 ± 23 pA/pF, n = 14; CeM-ES, 71 ± 12 pA/pF, n = 9; P < 0.05, Wilcoxon rank-sum test; Fig. 2j). Thus, unlike the CeL, the CeM showed differential ASIC expression in different subpopulations of GABAergic neurons.

The ICM_MV_ and ICM_MD_ are clusters of GABAergic neurons between the BLA and CeA, receiving input from the BLA and sending projections to the CeL and CeM (Fig. 1a)^39^. On the other hand, the lateral ICM (ICM_L_) is located on the dorsal–lateral side of the BLA, projecting inhibitory outputs to the BLA (Fig. 1a)^39^. We found that all ICM neurons showed similar electrophysiological properties (Supplementary Table S4) and exhibited modest ASIC currents similar to CeL neurons (ICM_MV_, 54 ± 8 pA/pF, n = 5; ICM_MD_, 44 ± 9 pA/pF, n = 6; ICM_L_, 54 ± 8 pA/pF, n = 5; Fig. 2k, l, m, and n).

In summary, the above functional mapping of ASIC current densities in diverse cell types of the amygdala network showed that ASICs were highly expressed in the major cell types of input (BLA) and output (CeM) regions of the amygdala (Fig. 2o).

### Differential expression of ASICs selectively contributed to LTP in amygdala circuits

**Figure 3:**
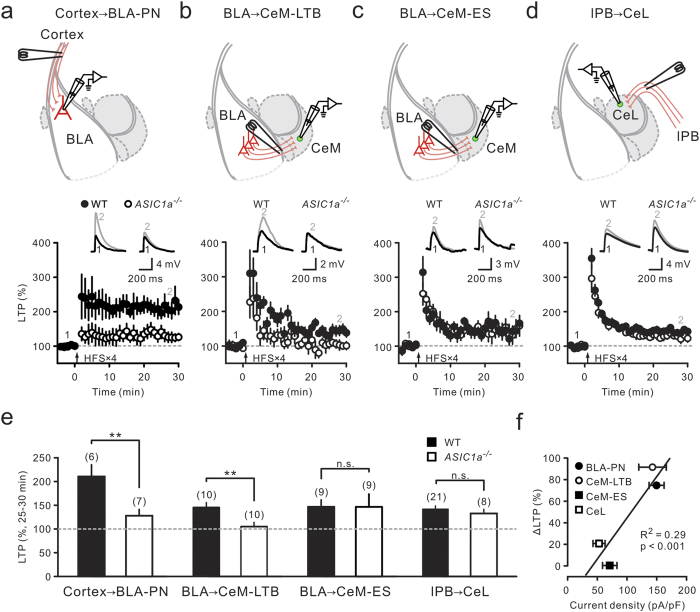
ASICs selectively contributed to LTP at glutamatergic synapses onto amygdala neurons. (**a**–**d**) Top, experimental configurations. Synaptic responses were measured for cortex→BLA-PN (**a**), BLA→CeM-LTB (**b**), BLA→CeM-ES (**c**), and lPB→CeL (**d**) neuron synapses. Bottom, averaged synaptic response (normalized) plotted against time. Representative averaged EPSP traces before (black) and after HFS (gray) were taken at the time indicated by number. (**e**) Summary of LTP magnitude, which was measured 25–30 min after HFS (arrow), at each synapse in WT and *ASIC1a*^–/–^ mice. ^**^P < 0.01; n.s., not significant, Wilcoxon rank-sum test. (**f**) Plot of the percentage of reduction in LTP magnitude versus ASIC current density of postsynaptic neurons. The percentage of LTP reduction correlates with ASIC current density.

The functional significance of distinctly high ASIC expression in the main input (BLA-PNs) and output (CeM-LTB) amygdala neurons was further examined by studying synaptic plasticity in these neurons. A recent study showed that ASIC1a contributes to LTP at cortical inputs to BLA-PNs, and that alterations in proton-ASIC signaling modulate the magnitude of LTP at this synapse^9^. Since fear conditioning causes widespread plasticity at multiple synapses in amygdala circuits^22^,^23^,^24^,^25^, we investigated whether ASIC expression contributed to LTP at glutamatergic synapses onto various types of amygdala neurons. Using a standard high-frequency stimulation (HFS) protocol, we compared LTP induction at glutamatergic synapses onto four distinct types of amygdala neurons (i.e., BLA-PN, CeM-LTB, CeM-ES, and CeL neurons) in brain slices from WT and *ASIC1a*^–/–^ (*Nestin*^*Cre/+*^; *ASIC1a*^*fl*/*fl*^) mice (Fig. 3a, b, c, and d, top). Consistent with a previous study^9^, we found that HFS evoked robust LTP (211 ± 25%; n = 6 cells, 4 animals; P < 0.05, Wilcoxon signed-rank test; Fig. 3a, bottom) at cortex→BLA-PN synapses in WT littermates, but small LTP (128 ± 13%; n = 7 cells, 5 animals; P = 0.15, Wilcoxon signed-rank test; Fig. 3a, bottom) in *ASIC1a*^–/–^ mutants. Interestingly, LTP evoked at BLA→CeM-LTB neuron synapses in WT neurons (144 ± 10%; n = 10 cells, 7 animals; P < 0.05, Wilcoxon signed-rank test; Fig. 3b, bottom) was also significantly larger than that in *ASIC1a*^–/–^ neurons (104 ± 9%; n = 10 cells, 6 animals; P = 0.92, Wilcoxon signed-rank test; Fig. 3b, bottom). In contrast, LTP evoked at BLA→CeM-ES neuron synapses in WT mice (147 ± 15%; n = 9 cells, 7 animals; P < 0.05, Wilcoxon signed-rank test; Fig. 3c, bottom) was not significantly different from that in *ASIC1a*^–/–^ mice (146 ± 27%; n = 9 cells, 6 animals; P = 0.08, Wilcoxon signed-rank test; Fig. 3c, bottom).

Glutamatergic inputs from the lateral parabrachial nucleus (lPB) in the brainstem also exhibit LTP^40^,^41^ and are required for conditioned fear learning and expression^41^. Similar to CeM-ES neurons, we found that LTP evoked at lPB→CeL neuron synapses in WT neurons (140 ± 7%; n = 21 cells, 16 animals; P < 0.001, Wilcoxon signed-rank test; Fig. 3d, bottom) was similar to that in *ASIC1a*^–/–^ neurons (132 ± 9%; n = 8 cells, 5 animals; P < 0.05, Wilcoxon signed-rank test; Fig. 3d, bottom).

Collectively, ASIC1a deletion caused a highly significant reduction in the magnitude of LTP in BLA-PNs and CeM-LTB neurons (Fig. 3e), both of which exhibited the highest ASIC current density, whereas this deletion had little effect on LTP in CeM-ES and CeL neurons that expressed relatively low levels of ASICs. This suggests that the level of ASIC expression contributed to the extent of LTP induction. In support of this idea, we found that the percentage of reduction in the LTP magnitude (ΔLTP%) in *ASIC1a*^–/–^ littermates, compared to WT mice, positively correlated with the ASIC current density observed in the postsynaptic neuron (Pearson’s R = 0.528; P < 0.001, F-test; Fig. 3f). Thus, the differential expression of ASICs among amygdala neurons contributed to LTP at both cortico-BLA and intra-amygdala glutamatergic synapses.

### ASIC1a deletion in GABAergic neurons selectively impaired LTP at BLA-CeM neuron synapses

**Figure 4:**
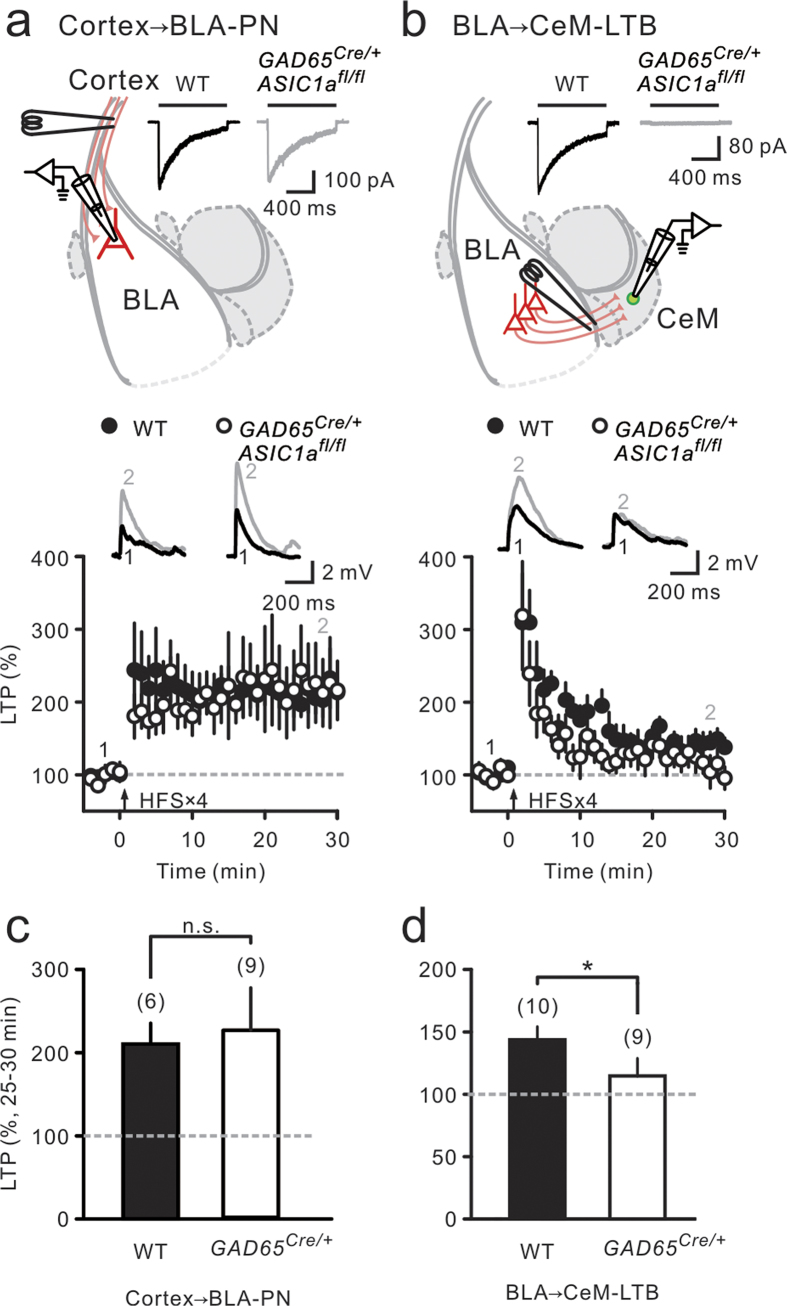
ASIC1a deletion in GABAergic neurons impaired LTP at CeM-LTB neuron synapses. (**a**, **b**) Top, experimental configurations. Representative ASIC current traces evoked in BLA-PN (**a**) and CeM-LTB neurons (**b**) from WT and mutant (*GAD65*^*Cre/+*^; *ASIC1a*^*fl/fl*^) mice. Black bars denote the application of pH 5 acidic solution. Bottom, synaptic responses were measured for cortex→BLA-PN (**a**) and BLA→CeM-LTB neuron (**b**) synapses. Averaged synaptic response (normalized) plotted against time. Representative averaged EPSP traces before (black) and after HFS (gray) were taken at indicated time points. (**c**–**d**) Summary of LTP magnitude, which is the average of the normalized postsynaptic responses at 25–30 min after HFS, at synapses in WT and mutant mice. ^*^P < 0.05; n.s., not significant, Wilcoxon rank-sum test.

Next, we investigated whether LTP at cortex→BLA-PN synapses was normal if ASICs were deleted in GABAergic neurons only. By crossing *ASIC1a*^*fl*/*fl*^ mice with *GAD65-Cre* transgenic mice, we generated GABAergic neuron-specific conditional deletion of ASIC1a in their offspring. Indeed, normal ASIC currents were detected in BLA-PNs (Fig. 4a, top), whereas ASIC currents were eliminated in CeM neurons (Fig. 4b, top) in mice (*GAD65*^*Cre/+*^; *ASIC1a*^*fl*/*fl*^) with specific ASIC1a deletion in GABAergic neurons. In line with this finding, we also found that the application of HFS robustly induced LTP at cortex-BLA-PN synapses in mutant mice (*GAD65*^*Cre/+*^; *ASIC1a*^*fl*/*fl*^), which had the specific ASIC1a deletion in GABAergic neurons (225 ± 51%; n = 9 cells, 5 animals; P < 0.01, Wilcoxon signed-rank test; Fig. 4a, bottom). On average, the magnitudes of LTP at cortex-BLA-PN synapses were similar in WT mice and mutants (Fig. 4c). Finally, we tested whether selective deletion of ASIC1a in GABAergic cells, including amygdala output neurons, eliminated LTP in these cells. We found that HFS failed to induce significant LTP (115 ± 14%; n = 9 cells, 5 animals; P = 0.30, Wilcoxon signed-rank test; Fig. 4b, bottom) at BLA-CeM-LTB neuron synapses in mutants (*GAD65*^*Cre/+*^; *ASIC1a*^*fl*/*fl*^), similar to no LTP in mice with pan-neuronal ASIC1a deletion (Fig. 3e; P = 0.60, Wilcoxon rank-sum test), but in contrast to marked LTP induction at the same synapse in WT mice (Fig. 4d).

### Deleting ASIC1a in GABAergic neurons impaired conditioned fear learning

The impairment of fear learning seen in ASIC1a-null mice could be prevented by specific transgenic expression of ASIC1a in the amygdala ^7^. The finding that disrupting ASIC1a in mice resulted in deficits in LTP at cortex→BLA-PN synapses has prompted the notion that ASIC-dependent LTP at the sensory inputs to the amygdala plays a critical role in conditioned fear learning^9^. In light of the present finding of ASIC-dependent LTP in the amygdala output neurons, we further examined whether deleting ASIC1a in GABAergic neurons also affected conditioned fear behavior. Using mice with either pan-neuronal ASIC1a deletion or a specific deletion of ASIC1a in GABAergic neurons, we next compared the behavioral effects of ASIC1a deletion in all neurons versus deletion in GABAergic neurons only.

**Figure 5:**
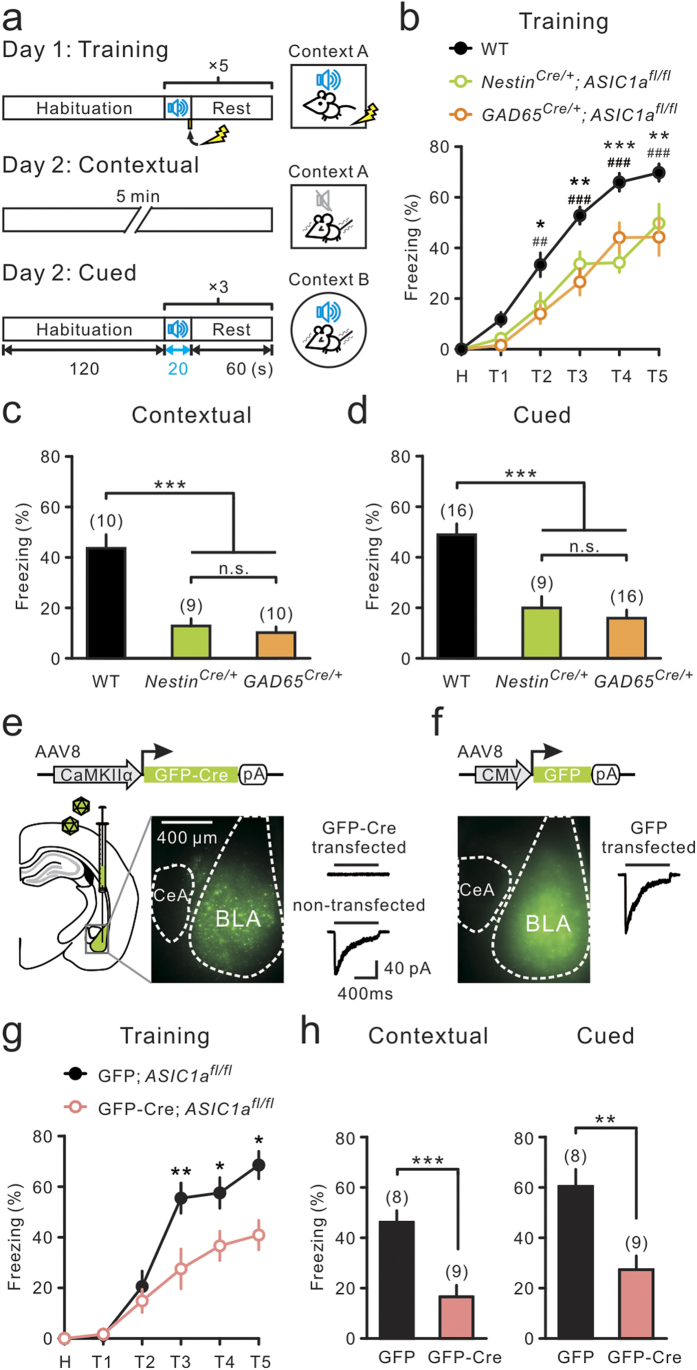
Deleting ASIC1a in GABAergic neurons impaired conditioned fear behavior. (**a**) Experimental protocol of auditory fear conditioning on day 1 and memory rec**a**ll tests on day 2. (**b**) Plot of relative time spent in freezing versus the trial (T) of fear conditioning. H, habituation. ^*^P < 0.05; ^**^P < 0.01; ^***^P < 0.001, WT versus *Nestin*^*Cre/ + *^; *ASIC1a*^*fl*/*fl*^; ^##^P < 0.01; ^###^P < 0.001, WT versus *GAD65*^*Cre/+*^; *ASIC1a*^*fl/fl*^, Wilcoxon rank-sum test. (**c**, **d**) Relative time spent in freezing during contextual and cued tests. ^***^P < 0.001, Wilcoxon rank-sum test. (**e**, **f**) AAV8 vector en**c**oding GFP-Cre or GFP was injected into BLA of *ASIC1a*^*fl/fl*^ mice. Left, epifluorescence images showing GFP-Cre and GFP expr**e**ssion at BLA. Right, representative ASIC currents recorded from transfected and non-transfected neurons. Black bars denote the application of pH 5 acidic solution. Scale bars in (**e**) apply to (**f**). (**g**) Plot of relative time spent in freezing versus the trial of fear conditioning. ^*^P < 0.05; ^**^P < 0.01, GFP-Cre; *ASIC1a*^*fl*/*fl*^ versus GFP; *ASIC1a*^*fl*/*fl*^, Wilcoxon rank-sum test. (**h**) Relative time spent in freezing during memory recall tests. ^***^P < 0.001, ^**^P < 0.01, Wilcoxon rank-sum test.

Pavlovian fear conditioning was used to test the role of ASICs in learned fear^1^,^2^. Mice were placed into the conditioning box (context A) and received five trials of tone-foot shock pairings. As illustrated (Fig. 5a), an initially neutral, auditory pure-tone conditioned stimulus (CS) was paired with a noxious unconditioned stimulus (US, an aversive footshock). During the training on day 1, we found that pan-neuronal ASIC deletion mice (*Nestin*^*Cre/+*^; *ASIC1a*^*fl*/*fl*^) showed decreased CS/US-induced freezing responses, relative to WT littermates (Fig. 5b), suggesting impaired fear learning in ASIC1a-deletion mice. Interestingly, mice with specific ASIC1a deletion in GABAergic neurons (*GAD65*^*Cre/+*^; *ASIC1a*^*fl*/*fl*^) also exhibited impaired learning behavior similar to that found in pan-neuronal ASIC1a-deletion mice (Fig. 5b). On day 2, context- and cue-dependent fear memory was tested. We found that presenting either the training context or the cue (tone) elicited normal high-level freezing responses in WT mice (Fig. 5c, d). However, mice with either pan-neuronal ASIC1a deletion or specific ASIC1a deletion in GABAergic neurons showed similar, but significantly lower, freezing levels (Fig. 5c, d).

Finally, we tested the behavioral impact of selective deletion of ASIC1a in glutamatergic PNs of the BLA, using the Cre/*loxP* system. We found that bilaterally injecting an adeno-associated viral vector serotype 8 (AAV8), encoding GFP-Cre under the control of the CaMKIIα promoter (AAV8-CaMKIIα-GFP-Cre), into the BLA of *ASIC1a*^*fl/fl*^ mice specifically eliminated ASIC currents in virus-transfected BLA-PNs, but not in non-transfected neurons (Fig. 5e) or neurons transduced with the fluorophore alone (Fig. 5f). Moreover, *ASIC1a*^*fl/fl*^ mice that received the viral vector encoding GFP-Cre in the BLA exhibited lower freezing levels than mice injected with a viral vector encoding GFP alone during conditioning (Fig. 5g) and the recall test (Fig. 5h). In summary, these results indicate that ASIC1a expression in GABAergic neurons was as important as ASIC1a expression in glutamatergic neurons in supporting conditioned fear behavior.

## Discussion

In this study, we characterized ASIC function in the amygdala at cellular, synaptic, and behavioral levels. Using *ASIC1a*^–/–^ and *ASIC2*^–/–^ mice, we found that ASIC1a and ASIC2 were co-expressed in the amygdala neurons. Moreover, ASIC expression level was highly variable and cell type-specific. In support of the notion that ASIC is a synaptic modulator, the extent of LTP correlated positively with the ASIC current density in the postsynaptic neuron. Importantly, ASIC1a deletion in GABAergic neurons only eliminated LTP of intra-amygdala glutamatergic synapses onto CeM output neurons and reduced both contextual and cued fear memory to the same extent as that found for ASIC1a deletion in all neurons or selectively in BLA glutamatergic PNs. Thus, fear learning requires ASIC-dependent LTP at multiple amygdala synapses, including both cortico-BLA input synapses, as well as intra-amygdala synapses on output neurons.

Our recent study shows differential expression of ASICs in different cell types within hippocampal networks^19^. Somatic ASIC current density of oriens lacunosum-moleculare (O-LM) cells in the CA1 region, a classical type of dendrite-targeting IN, is 6 times greater than that of fast-spiking basket cells (BCs) in the dentate gyrus, a major class of soma-targeting IN. Similarly, ASIC currents in dendrites of O-LM cells evoked by local acid puffing are approximately 6-fold greater than those in BC dendrites at remote distances (up to 100 μm) from the soma^19^. Nevertheless, there is a general concern whether somatic ASICs are equally representative of those in the synaptic membrane. In this study, a detailed characterization of ASICs was made from patches excised from somata rather than dendrites. Therefore, it is important to be aware of this potential pitfall in the interpretation of the correlation between the extent of LTP and the ASIC current density in the postsynaptic neurons. Finally, people should keep in mind that the extent of ASIC-dependent LTP might be sensitive to the patterns of induction paradigms used at synapses. In this study, we used the LTP induction protocol for each synapse type according to previous studies (see Methods).

It is intriguing to note that *GAD65*^*Cre/+*^; *ASIC1a*^*fl/fl*^ mice show a similar extent of fear learning/memory deficit as *Nestin*^*Cre/+*^; *ASIC1a*^*fl/fl*^ mice. The CeM is the final output station projecting to the brainstem areas^42^, which are responsible for freezing expression. The selective disruption of synaptic plasticity at the BLA→CeM-LTB synapses, but not cortex→BLA-PN synapses, in *GAD65*^*Cre/+*^; *ASIC1a*^*fl/fl*^ mice indicates a central role of the CeM in fear expression. Nevertheless, we are aware of the important role of BLA-INs in controlling fear learning^43^. Thus, we cannot exclude the possibility that ASIC1a deletion in GABAergic INs in *GAD65*^*Cre/+*^; *ASIC1a*^*fl/fl*^ mice causes abnormal inhibitory control in the BLA, given that ASIC expression levels are high in most BLA-IN subtypes. Finally, we speculate that similar changes in LTP induction can be observed in cortex-BLA-PN or BLA-CeM-LTB pathway induced by selective deletion of ASIC1a in glutamatergic neurons using the AAV-Cre/loxP system. However, if it is not the case, it implies that deletion of ASICs during early embryonic stages may disrupt synapse formation or alter the synaptic function during the development. If restoring ASIC1a with AAV-ASIC1a can rescues LTP in these two pathways in ASIC1a-null mice, it can exclude the essential role of ASIC during development.

The high level of ASIC expression in the primary input (i.e., BLA) and output (i.e., CeM) neurons of the fear circuitry is in accordance with the behavioral observation that ASIC1a deletion leads to deficits in fear acquisition and expression. In terms of fear expression, at least two types of CeM neurons project to two distinct brainstem sites: the periaqueductal gray and dorsal vagal complex^42^. With respect to brainstem-projecting neurons, whether there is a correspondence between the cell type (i.e., LTB versus ES) and their brainstem site remains unknown. On the other hand, information transfer from the lateral amygdala to the CeM is flexibly gated, depending on the specific pattern of environmental cues confronting the animal^44^. It is thought that the CeL and ICMs fulfill this function, as they receive glutamatergic inputs from the BLA and send GABAergic projections to the CeM^3^. As described above, ASICs are modestly expressed in CeL and ICM neurons. The role of ASICs in these neurons and their relative contribution to the acquisition of fear conditioning and expression of conditioned fear require further studies, for which cell type-specific gene disruption, as demonstrated here, would prove useful.

The gating properties of ASICs described here including desensitization and recovery time course (Supplementary Fig. S3) are similar to those of CA1 pyramidal neurons, which contain a mixture of homomeric ASIC1a, heteromeric ASIC1a/2a, and heteromeric ASIC1a/2b channels^15^,^19^. PcTX1 is known to inhibit ASIC1a homomers and ASIC1a/2b heteromers, but not ASIC1a/2a heteromers^33^. Therefore, the great majority (approximately 80%) of ASICs in amygdala neurons is likely mediated by ASIC1a/2a heteromers. Conversely, the PcTX1-sensitive component (approximately 20%) is mediated by ASIC1a homomers and/or ASIC1a/2b heteromers. Notably, the current (putatively mediated by ASIC1a homomers) in *ASIC2*^-/-^ mice, which is approximately 20% of the total current in WT mice, exhibits a rapid cumulative reduction in responses to successive applications of proton. This phenomenon is termed tachyphylaxis^45^. Since only ASIC1a homomer exhibits prominent tachyphylaxis^45^, we speculate that PcTX1-sensitive component is primarily mediated by ASIC1a homomers. The difference in ASIC current densities in various cell types reflects the variation in the expression of ASIC subunits. In our study, since no difference in gating properties was found in ASIC currents recorded from all cell types (Supplementary Fig. S3), the differential expression of ASICs in each cell type may involve the coordinated regulation of ASIC subunit expression, without altering the relative contribution by ASIC1a homomer, and ASIC1a/2b and ASIC1a/2a heteromers.

Two recent studies showed that ASICs participate in synaptic transmission in the amygdala and nucleus accumbens where they contribute to approximately 5% of excitatory postsynaptic currents^9^,^46^. ASICs are Na^+^- and Ca^2+^-permeable channels that are activated by extracellular acidosis^29^. We speculate an important role of proton-ASIC signaling during intense presynaptic stimulation. During basal transmission, synaptic pH drop is rapidly buffered by a variety of homeostatic mechanisms^47^. Massive extracellular pH reductions may occur during intense activity such as fear conditioning. The lower pH can generate larger ASIC currents, thereby boosting *N*-methyl-d-aspartate (NMDA) receptor function^18^. These effects could explain the involvement of protons and the requirement of ASICs for LTP induced by HFS. In this scenario, the strength of proton-ASIC signaling should be critical in determining the relative contribution of ASICs to learning, memory, and synaptic plasticity. Indeed, several lines of evidence support this view. First, ASIC1a and ASIC2 expression in the hippocampus is relatively low, compared to other brain regions^5^,^20^. Consistent with this, *ASIC1a*^–/–^, *ASIC2*^–/–^, and *ASIC1a/2*^–/–^ mice display normal spatial learning and memory^20^,^48^. Second, ASIC currents during synaptic transmission are not detected in the hippocampus. In keeping with this, LTP at CA3-CA1 synapses can be readily induced in *ASIC1a*^–/–^ mice^48^. Third, the observed effects of ASICs on synaptic plasticity, learning, and behavior are found in the amygdala and nucleus accumbens, the brain regions where ASICs are highly enriched^9^,^46^. Fourth, increasing or decreasing pH buffering capacity can bidirectionally modulate the magnitude of LTP at cortex→BLA-PN synapses^9^. Finally, in our study, the reduction in the magnitude of LTP at different glutamatergic synapses in *ASIC1a*^–/–^ mice, relative to WT controls, correlates positively with the abundance of ASICs in postsynaptic neurons (Fig. 3f). Collectively, our study also supports the view that postsynaptic ASIC is a positive regulator of associative fear learning and memory at both the synaptic and behavioral levels. Such knowledge could be of importance for future drug discovery and development.

A recent study finds that disrupting ASIC1a leads to an increase in spine density and changes in glutamate receptor function in nucleus accumbens, although the underlying mechanisms remain unknown^46^. Thus, it is worth to note that our study did not exclude the possibility that ASICs are involved in regulation of synapse structure and function in the amygdala neurons. Further experiments are needed to investigate whether ASIC is required for LTP in a cell-autonomous fashion. Several outstanding questions remain to be answered, including why ASIC is important for LTP at some certain synapses, and why ASICs are so enriched in several BLA-IN subtypes, but not in FSINs. Furthermore, it will be of interest to determine whether ASIC mediates or modulates synaptic plasticity in BLA-INs. Finally, the striking differences in the ASIC expression level between non-FSINs and FSINs raise general questions regarding the roles of ASIC in amygdala INs in fear learning and memory.

## Methods

### Brain slice preparation

Coronal brain slices (300 μm thick) were prepared from mice (19–75 days old for AISC characterization; 1–4 months old for LTP experiments and 3–5 months old for behavioral tests) of either sex on C57BL/6 genetic background, including WT, *GAD67-GFP* KI, *ASIC1a*^–/–^, and *ASIC2*^–/–^ mice, using a vibratome (DTK-1000, Dosaka, Kyoto, Japan), as described previously^49^. Animals were killed by decapitation, in accordance with national and institutional guidelines, and all procedures were approved by the Animal Care and Use Committee of National Yang-Ming University. Floxed ASIC1a (*ASIC1a*^*fl/fl*^) mice were crossed with *Nestin-Cre* and *GAD65-Cre* transgenic mice to generate *ASIC1a* gene deletion in their offspring^48^. *ASIC2*^–/–^ mice^50^ were obtained from Jackson Laboratory (Bar Harbor, ME, USA). To facilitate cell type identification, we used *GAD67-GFP* KI mice, in which GFP expression was driven by the GAD67 promoter^27^. Slices were sectioned in ice-cold cutting solution containing (in mM): 87 NaCl, 25 NaHCO_3_, 1.25 NaH_2_PO_4_, 2.5 KCl, 10 glucose, 75 sucrose, 0.5 CaCl_2_ and 7 MgCl_2_. Following sectioning, slices were incubated in the cutting solution (oxygenated with 95% O_2_/5% CO_2_) in a holding chamber at 34 °C for 30 min, and then at room temperature until used. During experiments, individual slices were transferred to a submersion recording chamber and were continuously superfused with oxygenated artificial cerebrospinal fluid (CSF) containing (in mM): 125 NaCl, 25 NaHCO_3_, 1.25 NaH_2_PO_4_, 2.5 KCl, 25 glucose, 2 CaCl_2_ and 1 MgCl_2_. The recording temperature was 25 ± 3 °C. Neurons were visualized under the guidance of infrared differential interference contrast (DIC) optics and by their green fluorescence with epifluorescence illumination.

### Nucleated patch recordings

Nucleated patch recordings were made, as described previously^49^,^51^, using an Axopatch 200B amplifier (Molecular Devices, Sunnyvale, CA, USA). Pipette capacitance was compensated. Signals were low-pass filtered at 5 kHz (four-pole Bessel) and sampled at 10 kHz using a digitizer (Digidata 1440 A; Molecular Devices). Pulse sequences were generated by a Digidata 1440 A via pClamp 10.2 (Molecular Devices). Minor and major axes of nucleated patches were measured. It was assumed that nucleated patches were approximately ellipsoid^52^, and the membrane surface area was calculated using the following formula:


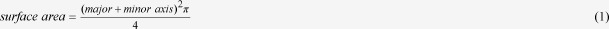


The total membrane capacitance was determined from the surface area, using the value (1 μF/cm^2^) of specific membrane capacitance^52^,^53^. Fast application of H^+^ on nucleated patches, isolated from identified neurons, was performed, as described previously^19^. Fast application experiments were started 1–2 min after the patches were excised. Double-barreled application pipettes were fabricated from theta glass capillaries (2 mm outer diameter, 0.3 mm wall thickness, 0.12 mm septum, Hilgenberg GmbH, Malsfeld, Germany) and mounted on a piezoelectric-based solution switching system (MXPZT-300, Siskiyou, OR, USA). The time necessary for the complete exchange of solutions was determined using an open patch pipette by switching between Na^+^-rich and 10% Na^+^-rich solutions, which was 186 ± 14 μs (n = 3) by measuring 20–80% rise time of the junction potential change. ASIC currents evoked by 1 s pulses of H^+^ were applied every 20 s, except for some pharmacological experiments where short pulses (50 ms) were used.

To evoke ASIC currents with various pH values, 2-(*N*-morpholino)ethanesulfonic acid (MES)-buffered Na^+^-rich external solution in the test barrel was used, containing (in mM): 135 NaCl, 5.4 KCl, 1.8 CaCl_2_, 1 MgCl_2_, 10 MES, adjusted to the desired values with *N*-methyl-d-glucamine. The intracellular solution contained (in mM): 142 K-gluconate, 2 KCl, 0.2 EGTA, 4 MgATP, 10 HEPES, 7 Na_2_-phosphocreatine; pH adjusted to 7.3 with KOH. Bovine serum albumin (0.1%) was added to external solutions containing the spider toxin PcTX1 (Peptides International, Louisville, KY, USA) to prevent its absorption into tubing and containers. For PcTX1 application, both pH 7.4 and 5 external solutions contained 30 nM PcTX1. The nucleated patches were placed in the pH 7.4 solution at least 1 min before the fast-application experiment. All other chemicals were from Sigma (St. Louis, MO, USA), unless specified otherwise.

### Measurement of synaptic responses and LTP induction

Excitatory postsynaptic potentials (EPSPs) or currents (EPSCs), in the presence of GABA receptor type A (GABA_A_R) antagonist gabazine (1 μM), were recorded from identified neurons in current or voltage clamp. Basal synaptic responses were evoked by a brief pulse (0.1–0.5 ms) of either constant voltage or current delivered by a stimulus isolation unit (Isoflex, A.M.P.I., Jerusalem, Israel) every 20 s with tungsten bipolar electrodes. Stimulation electrodes were positioned at axonal bundles of inputs of interest. Synaptic strength was quantified as the peak amplitude of EPSP or EPSC. Baseline responses were collected with a stimulation intensity that yielded 10–30% of the maximal response. Slices displaying unstable baseline recording or series resistance change of >20% were discarded. For the cortex→BLA-PN synapse, LTP was induced by pairing four trains of HFS (HFS × 4, 100 Hz for 1 s per train; 10 s intervals) with brief supra-threshold current step injections (approximately 2 nA; 1 ms) into the postsynaptic cell (with a 5 ms delay) in a current clamp. For the BLA→CeM neuron synapse, LTP was induced by HFS × 4 (10 s intervals), with postsynaptic cells held at –70 mV in a current clamp^54^. Similarly, HFS × 4 (20 s intervals), with postsynaptic cells held at –50 mV, was used to induce LTP at the lPB→CeL neuron synapse^40^.

### Immunohistochemistry

For immunofluorescent labeling, brains were removed from *GAD67-GFP* KI mice which were transcardially perfused with 4% paraformaldehyde in 0.1 M phosphate buffered saline (PBS). Brains were further dehydrated in 15% sucrose for 24 h, and 30% sucrose for 24 h. Cryostat sections (20 μm thick) were rehydrated with PBS. Following a PBS wash, sections were permeabilized with 0.3% PBST (0.3 % triton-X-100 in PBS) for 30 min and incubated in 10% normal goat serum (NGS) for 2 h to block non-specific binding. To stain NeuN, which is a neuron-specific protein, sections were incubated in primary mouse anti-NeuN antibody (1:400; MAB377; Merck Millipore, Billerica, MA, USA) with 5% NGS in 0.3% PBST for 24 h at 4 °C. Following a PBS wash, sections were incubated in secondary antibody (goat anti-mouse Alexa Fluor 594; Life Technologies, Grand Island, NY, USA) with 2% NGS in 0.3% PBST for 2 h at 4 °C. Following a PBS wash, sections were mounted in VECTASHIELD HardSet Mounting Medium (Vector Laboratories, Burlingame, CA, USA) and viewed under a Zeiss Microscope Axio Observer A1 (Zeiss, Oberkochen, Germany).

### Fear conditioning

Fear conditioning was performed in two different contexts. On day 1, mice were placed into the conditioning box (context A) for 2 min habituation and then received five trials of tone (80 dB, 20 s)-foot shock (0.6 mA, 2 s) pairings. A foot shock was given 2 s before the end of a tone. There was 1 min observation time between each pairing. To evaluate fear learning, we recorded the freezing time during habituation and between each trial. The context-dependent fear memory test was performed 24 h later by re-exposing the mice for 5 min to context A. One hour later, mice were placed into context B for 2 min, which was followed by three trials of 20 s tone plus 1 min observation time to evaluate the tone-dependent fear memory. We recorded the freezing time to context or tone during the total observation time. Freezing is defined as a lack of movement (>2 s) associated with a crouching posture, except for heartbeat and respiration. The freezing time is expressed as a percentage of the total observation time.

### Virus injections

Mice (2–4 months old) were anaesthetized with isoflurane and placed into a stereotaxic frame (Stoelting, Wood Dale, IL, USA), with the mouths and noses covered by the anaesthetizing mask with constant air flow containing 1.5% isoflurane (air flow rate: 4 mL/min). The mice were placed over a homeothermic blanket (Panlab Harvard apparatus, Barcelona, Spain) to keep their body temperature constant (36 °C), with their eyes protected by ophthalmic gel during securing. The skull was surgically exposed using scissors and drilled over the desired coordinates. AAV8-CMV-GFP was generated in the laboratory of our collaborator Dr. Min-Hong Tai (National Sun Yat-Sen University, Taiwan). The transgene (pAAV-CMV-GFP), packaging (pLT-RC08), and helper (pHGTI-adeno1) constructs were gifts from Dr. Jeng-Shin Lee (Harvard Gene Therapy Initiative, Harvard Medical School, Boston, MA, USA). AAV2/8 was serotyped with AAV8 capsid proteins. The AAV8-CaMKIIα-GFP-Cre was purchased from University of North Carolina Vector Core (Chapel Hill, NC, USA). The virus was delivered through the craniotomy bilaterally using a 10 μL NanoFil syringe (World Precision Instruments, Sarasota, FL, USA) and a 35-gauge beveled metal needle. The injection volume (0.5 μL at each location) and flow rate (0.1 μL/min) were controlled with a nanopump Controller (KD Scientific, Holliston, MA, USA). The needles were left in position for 10 min after injection. Injection coordinates, relative to Bregma, were: anterior–posterior, –2 mm; medial–lateral, ±3.4 mm; dorsal–ventral, –5 mm and –5.1 mm. Mice were allowed to recover for 3 weeks, following injection.

### Hierarchical clustering analysis

For cell classification, unsupervised clustering^34^,^55^,^56^ was performed, using squared Euclidean distances and Ward’s method^36^. Electrophysiological properties of BLA INs and CeL neurons were tested for uniformity in their distributions. Variables with a nonuniform distribution (see Supplementary Fig. S1a, S1b, S1c, S1d, S2c, S2d, S2e, and S2f) were used for subsequent unsupervised clustering. Hierarchical clustering was operated, as follows. First, each neuron was transformed into a four-dimensional data point with variables. Before clustering, variables were first normalized into the range (0, 1) by performing Min-max normalization. The distance between data points represented the dissimilarity between them; closer data points have higher similarity. Next, all the data points were clustered by the following iterative procedure. First, each data point was assigned to a cluster; every cluster therefore contained only one data point. Second, the two closest clusters were merged into one cluster, such that there would be one fewer cluster. Third, the distance between the new cluster and each of the old clusters was determined. Fourth, steps two and three were repeated, until there was only one cluster left. Ward’s method linkage rules^36^ minimize the error sum of squares of any pair of cluster in step three. The pair of clusters with a minimum between-cluster distance was merged. The hierarchical clustering analysis was carried out using Free Statistics Software v.1.1.23-r7. (Wessa, P. *Free statistics software,* available at: http://www.wessa.net/)

### Data analysis and statistics

Data were analyzed and fitted with Clampfit 10.2 (Molecular Devices) and Prism 5.01 (GraphPad, La Jolla, CA, USA). The accommodating ratio is the maximal ratio of the mean of the last five inter-spike intervals (ISIs) divided by the mean of the first five ISIs under minimal current step injection (<400 pA) which could generate>10 APs per second. The spike delay is the latency of the first AP upon 1 s near-threshold depolarizing (rheobase) current step injection. The ISI ratio is calculated as the latter ISI divided by the previous ISI. The maximal coefficient of variation (CV) of ISI ratios was calculated from spike trains evoked by 1 s current step injection. The maximal mean firing rate is the maximal number of APs that could be generated by 1 s current step injection. Input resistance is defined by the ratio of steady-state voltage change/1 s hyperpolarizing current (–25 pA or –50 pA). For classification of BLA GFP-expressing cells, four features consisting of the accommodating ratio, maximal CV of ISI ratio, delay, and maximal mean firing rate were used to identify different cell types (Supplementary Fig. S1). The desensitization time constant of ASIC current was obtained by fitting currents with the function shown below:


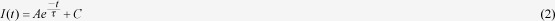


*A* denotes the peak amplitude of current, *τ* represents the desensitization time constant, and *C* denotes the amplitude of steady-state current.

The pH-response curve was fitted with the function, as shown below:


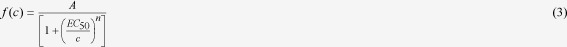


where *A* is the constant for the maximal effect, *c* denotes the concentration, *EC*_50_ represents the half-maximal effective concentration, and *n* denotes the Hill coefficient.

For reversal potential measurements, data points of *I*-*V* relations were fitted with second-order polynomials, from which the interpolated potentials were calculated. The theoretical reversal potential of sodium channels (*E*_Na_) was calculated, according to the Nernst equation:


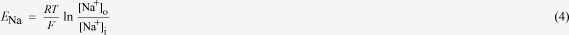


where [Na^+^]_o_, [Na^+^]_i_ are outer and inner Na^+^ concentrations, and *F*, *R*, *T* have standard thermodynamic meanings^57^.

Values indicate mean±s.e.m. (standard error of mean); error bars in figures also represent s.e.m. Statistical significance among groups was tested using the nonparametric Kruskal–Wallis test. Where significant, pairwise comparisons, using the Wilcoxon rank-sum test, were carried out for each pair of groups. Statistical significance of linear regression was tested using the F-test. All tests were performed at the significance level (P) as indicated. The s.e.m. of reversal potentials were obtained by analyzing data of individual experiments separately.

## Author Contributions

P.-H. C. performed most of the experiments. T.-C. C. carried out a subset of LTP and behavioral experiments. Y. Y., C.-C. C, and C.-C. L. designed the experiments. C.-C. L. and P.-H. C. wrote the manuscript.

## Additional Information

**How to cite this article**: Chiang, P.-H. *et al*. ASIC-dependent LTP at multiple glutamatergic synapses in amygdala network is required for fear memory. *Sci. Rep.* **5**, 10143; doi: 10.1038/srep10143 (2015).

## Supplementary Material

Supplementary Information

## References

[b1] LeDoux J. E. Emotion circuits in the brain. Annu. Rev. Neurosci. 23, 155–184 (2000). 10845062 10.1146/annurev.neuro.23.1.155

[b2] Ehrlich I. *et al.* Amygdala inhibitory circuits and the control of fear memory. Neuron 62, 757–771 (2009). 19555645 10.1016/j.neuron.2009.05.026

[b3] Duvarci S. & Paré D. Amygdala microcircuits controlling learned fear. Neuron 82, 966–980 (2014). 24908482 10.1016/j.neuron.2014.04.042 PMC4103014

[b4] Smoller J. W. *et al.* The human ortholog of acid-sensing ion channel gene ASIC1a is associated with panic disorder and amygdala structure and function. Biol. Psychiatry 76, 902–910 (2014). 24529281 10.1016/j.biopsych.2013.12.018 PMC4103972

[b5] Wemmie J. A. *et al.* Acid-sensing ion channel 1 is localized in brain regions with high synaptic density and contributes to fear conditioning. J. Neurosci. 23, 5496–5502 (2003). 12843249 10.1523/JNEUROSCI.23-13-05496.2003 PMC6741257

[b6] Coryell M. W. *et al.* Targeting ASIC1a reduces innate fear and alters neuronal activity in the fear circuit. Biol. Psychiatry 62, 1140–1148 (2007). 17662962 10.1016/j.biopsych.2007.05.008

[b7] Coryell M. W. *et al.* Restoring acid-sensing ion channel-1a in the amygdala of knock-out mice rescues fear memory but not unconditioned fear responses. J. Neurosci. 28, 13738–13741 (2008). 19091964 10.1523/JNEUROSCI.3907-08.2008 PMC2651157

[b8] Wemmie J. A. *et al.* Overexpression of acid-sensing ion channel 1a in transgenic mice increases acquired fear-related behavior. Proc. Natl. Acad. Sci. USA 101, 3621–3626 (2004). 14988500 10.1073/pnas.0308753101 PMC373512

[b9] Du J. *et al.* Protons are a neurotransmitter that regulates synaptic plasticity in the lateral amygdala. Proc. Natl. Acad. Sci. USA 111, 8961–8966 (2014). 24889629 10.1073/pnas.1407018111 PMC4066526

[b10] Price M. P., Snyder P. M. & Welsh M. J. Cloning and expression of a novel human brain Na^+^ channel. J. Biol. Chem. 271, 7879–7882 (1996). 8626462 10.1074/jbc.271.14.7879

[b11] Waldmann R., Champigny G., Bassilana F., Heurteaux C. & Lazdunski M. A proton-gated cation channel involved in acid-sensing. Nature 386, 173–177 (1997). 9062189 10.1038/386173a0

[b12] Waldmann R. & Lazdunski M. H^+^-gated cation channels: neuronal acid sensors in the NaC/DEG family of ion channels. Curr. Opin. Neurobiol. 8, 418–424 (1998). 9687356 10.1016/s0959-4388(98)80070-6

[b13] Lingueglia E. Acid-sensing ion channels in sensory perception. J. Biol. Chem. 282, 17325–17329 (2007). 17430882 10.1074/jbc.R700011200

[b14] Boiko N., Kucher V., Wang B. & Stockand J. D. Restrictive expression of acid-sensing ion channel 5 (Asic5) in unipolar brush cells of the vestibulocerebellum. PLoS ONE 9, e91326 (2014). 24663811 10.1371/journal.pone.0091326 PMC3963869

[b15] Baron A., Waldmann R., & Lazdunski M. ASIC-like, proton-activated currents in rat hippocampal neurons. J. Physiol. 539, 485–494 (2002). 11882680 10.1113/jphysiol.2001.014837 PMC2290154

[b16] Alvarez de la Rosa D. *et al.* Distribution, subcellular localization and ontogeny of ASIC1 in the mammalian central nervous system. J. Physiol. 546, 77–87 (2003). 12509480 10.1113/jphysiol.2002.030692 PMC2342460

[b17] Baron A., Voilley N., Lazdunski M. & Lingueglia E. Acid sensing ion channels in dorsal spinal cord neurons. J. Neurosci. 28, 1498–1508 (2008). 18256271 10.1523/JNEUROSCI.4975-07.2008 PMC6671562

[b18] Wemmie J. A. *et al.* The acid-activated ion channel ASIC contributes to synaptic plasticity, learning, and memory. Neuron 34, 463–477 (2002). 11988176 10.1016/s0896-6273(02)00661-x

[b19] Weng J. Y., Lin Y. C. & Lien C. C. Cell type-specific expression of acid-sensing ion channels in hippocampal interneurons. J. Neurosci. 30, 6548–6558 (2010). 20463218 10.1523/JNEUROSCI.0582-10.2010 PMC6632567

[b20] Price M. P. *et al.* Localization and behaviors in null mice suggest that ASIC1 and ASIC2 modulate responses to aversive stimuli. Genes Brain Behav. 13, 179–194 (2014). 24256442 10.1111/gbb.12108 PMC3998777

[b21] Sah P., Faber E. S., Lopez de Armentia M. & Power J. The amygdaloid complex: anatomy and physiology. Physiol. Rev. 83, 803–834 (2003). 12843409 10.1152/physrev.00002.2003

[b22] Humeau Y. *et al.* A pathway-specific function for different AMPA receptor subunits in amygdala long-term potentiation and fear conditioning. J. Neurosci. 27, 10947–10956 (2007). 17928436 10.1523/JNEUROSCI.2603-07.2007 PMC6672841

[b23] Sah P., Westbrook R. F. & Lüthi A. Fear conditioning and long-term potentiation in the amygdala: what really is the connection? Ann. N. Y. Acad. Sci. 1129, 88–95 (2008). 18591471 10.1196/annals.1417.020

[b24] Fourcaudot E. *et al.* L-type voltage-dependent Ca^2+^ channels mediate expression of presynaptic LTP in amygdala. Nat. Neurosci. 12, 1093–1095 (2009). 19648911 10.1038/nn.2378

[b25] Duvarci S., Popa D. & Paré D. Central amygdala activity during fear conditioning. J. Neurosci. 31, 289–294 (2011). 21209214 10.1523/JNEUROSCI.4985-10.2011 PMC3080118

[b26] Paré D. & Duvarci S. Amygdala microcircuits mediating fear expression and extinction. Curr. Opin. Neurobiol. 22, 717–723 (2012). 22424846 10.1016/j.conb.2012.02.014 PMC3380167

[b27] Tamamaki N. *et al.* Green fluorescent protein expression and colocalization with calretinin, parvalbumin, and somatostatin in the GAD67-GFP knock-in mouse. J. Comp. Neurol. 467, 60–79 (2003). 14574680 10.1002/cne.10905

[b28] McDonald A. J. Cytoarchitecture of the central amygdaloid nucleus of the rat. J. Comp. Neurol. 208, 401–418 (1982). 7119168 10.1002/cne.902080409

[b29] Lin Y. C., Liu Y. C., Huang Y. Y. & Lien C. C. High-density expression of Ca^2+^-permeable ASIC1a channels in NG2 glia of rat hippocampus. PloS ONE 5, e12665 (2010). 20844750 10.1371/journal.pone.0012665 PMC2937019

[b30] Askwith C. C., Wemmie J. A., Price M. P., Rokhlina T. & Welsh M. J. Acid-sensing ion channel 2 (ASIC2) modulates ASIC1 H^+^-activated currents in hippocampal neurons. J. Biol. Chem. 279, 18296–18305 (2004). 14960591 10.1074/jbc.M312145200

[b31] Zha X. M. *et al.* ASIC2 subunits target acid-sensing ion channels to the synapse via an association with PSD-95. J. Neurosci. 29, 8438–8446 (2009). 19571134 10.1523/JNEUROSCI.1284-09.2009 PMC2734339

[b32] Chen X., Kalbacher H. & Gründer S. The tarantula toxin psalmotoxin 1 inhibits acid-sensing ion channel (ASIC) 1a by increasing its apparent H^+^ affinity. J. Gen. Physiol. 126, 71–79 (2005). 15955877 10.1085/jgp.200509303 PMC2266618

[b33] Sherwood T. W., Lee K. G., Gormley M. G. & Askwith C. C. Heteromeric acid-sensing ion channels (ASICs) composed of ASIC2b and ASIC1a display novel channel properties and contribute to acidosis-induced neuronal death. J. Neurosci. 31, 9723–9734 (2011). 21715637 10.1523/JNEUROSCI.1665-11.2011 PMC3160670

[b34] Sosulina L., Graebenitz S. & Pape H. C. GABAergic interneurons in the mouse lateral amygdala: a classification study. J. Neurophysiol. 104, 617–626 (2010). 20484532 10.1152/jn.00207.2010

[b35] Song. C. *et al.* Stuttering interneurons generate fast and robust inhibition onto projection neurons with low capacity of short term modulation in mouse lateral amygdala. PLoS ONE 8, e60154 (2013). 23527307 10.1371/journal.pone.0060154 PMC3602081

[b36] Ward J. H. Jr. Hierarchical grouping to optimize an objective function. J. Am. Stat. Assoc. 58, 236–244 (1963).

[b37] Haubensak W. *et al.* Genetic dissection of an amygdala microcircuit that gates conditioned fear. Nature 468, 270–276 (2010). 21068836 10.1038/nature09553 PMC3597095

[b38] Li H. *et al.* Experience-dependent modification of a central amygdala fear circuit. Nat. Neurosci. 16, 332–339 (2013). 23354330 10.1038/nn.3322 PMC3581751

[b39] Likhtik E., Popa D., Apergis-Schoute J., Fidacaro G. A. & Paré D. Amygdala intercalated neurons are required for expression of fear extinction. Nature 454, 642–645 (2008). 18615014 10.1038/nature07167 PMC2528060

[b40] López de Armentia M. & Sah P. Bidirectional synaptic plasticity at nociceptive afferents in the rat central amygdala. J. Physiol. 581, 961–970 (2007). 17379642 10.1113/jphysiol.2006.121822 PMC2170827

[b41] Watabe A. M. *et al.* Synaptic potentiation in the nociceptive amygdala following fear learning in mice. Mol. Brain. 6, 11 (2013). 23452928 10.1186/1756-6606-6-11 PMC3606120

[b42] Viviani D. *et al.* Oxytocin selectively gates fear responses through distinct outputs from the central amygdala. Science 333, 104–107 (2011). 21719680 10.1126/science.1201043

[b43] Wolff S. B. *et al.* Amygdala interneuron subtypes control fear learning through disinhibition. Nature 509, 453–438 (2014). 24814341 10.1038/nature13258

[b44] Paré D., Royer S., Smith Y. & Lang E. J. Contextual inhibitory gating of impulse traffic in the intra-amygdaloid network. Ann. N. Y. Acad. Sci. 985, 78–91 (2003). 12724150 10.1111/j.1749-6632.2003.tb07073.x

[b45] Chen X. & Gründer S. Permeating protons contribute to tachyphylaxis of the acid-sensing ion channel (ASIC) 1a. J. Physiol. 579, 657–670 (2007). 17204502 10.1113/jphysiol.2006.120733 PMC2151377

[b46] Kreple C. J. *et al.* Acid-sensing ion channels contribute to synaptic transmission and inhibit cocaine-evoked plasticity. Nat. Neurosci. 17, 1083–1091 (2014). 24952644 10.1038/nn.3750 PMC4115047

[b47] Chesler M. & Kaila K. Modulation of pH by neuronal activity. Trends Neurosci. 15, 396–402 (1992). 1279865 10.1016/0166-2236(92)90191-a

[b48] Wu P. Y. *et al.* Acid-sensing ion channel-1a is not required for normal hippocampal LTP and spatial memory. J. Neurosci. 33, 1828–1832 (2013). 23365222 10.1523/JNEUROSCI.4132-12.2013 PMC6619135

[b49] Lien C. C., Martina M., Schultz J. H., Ehmke H., & Jonas P. Gating, modulation and subunit composition of voltage-gated K^+^ channels in dendritic inhibitory interneurones of rat hippocampus. J. Physiol. 538, 405–419 (2002). 11790809 10.1113/jphysiol.2001.013066 PMC2290075

[b50] Price M. P. *et al.* The mammalian sodium channel BNC1 is required for normal touch sensation. Nature 407, 1007–1011 (2000). 11069180 10.1038/35039512

[b51] Lien C. C. & Jonas P. Kv3 potassium conductance is necessary and kinetically optimized for high-frequency action potential generation in hippocampal interneurons. J. Neurosci. 23, 2058–2068 (2003). 12657664 10.1523/JNEUROSCI.23-06-02058.2003 PMC6742035

[b52] Gentet L. J., Stuart G.J. & Clements J.D. Direct measurement of specific membrane capacitance in neurons. Biophys. J. 79, 314–320 (2000). 10866957 10.1016/S0006-3495(00)76293-X PMC1300935

[b53] Chan C. F. *et al.* Ba^2+^- and bupivacaine-sensitive background K^+^ conductances mediate rapid EPSP attenuation in oligodendrocyte precursor cells. J. Physiol. 591, 4843–4858 (2013). 23940377 10.1113/jphysiol.2013.257113 PMC3800458

[b54] Fu Y. *et al.* Long-term potentiation (LTP) in the central amygdala (CeA) is enhanced after prolonged withdrawal from chronic cocaine and requires CRF_1_ receptors. J. Neurophysiol. 97, 937–941 (2007). 17079348 10.1152/jn.00349.2006

[b55] Cauli B. *et al.* Classification of fusiform neocortical interneurons based on unsupervised clustering. Proc. Natl. Acad. Sci. USA 97, 6144–6149 (2000). 10823957 10.1073/pnas.97.11.6144 PMC18572

[b56] Jasnow A. M., Ressler K. J., Hammack S. E., Chhatwal J. P. & Rainnie D. G. Distinct subtypes of cholecystokinin (CCK)-containing interneurons of the basolateral amygdala identified using a CCK promoter-specific lentivirus. J. Neurophysiol. 101, 1494–1506 (2009). 19164102 10.1152/jn.91149.2008 PMC2666417

[b57] Hille B. Ion channels of excitable membranes. (Sinauer Associates 2001).

