# Supplementary material for: ASIC-dependent LTP at multiple glutamatergic synapses in amygdala network is required for fear memory
Source: Sci Rep. 2015 May 19;5:10143. doi: 10.1038/srep10143 (PMC4437300; doi:10.1038/srep10143)
Supplement: Supplementary Information [file srep10143-s1.pdf]

Scientific Reports
Supplemental Information

ASIC-dependent LTP at multiple glutamatergic synapses in amygdala network is required for fear memory

Po-Han Chiang, Ta-Chun Chien, Chih-Cheng Chen, Yuchio Yanagawa and Cheng-Chang Lien

Supplemental Information

Supplemental Figures

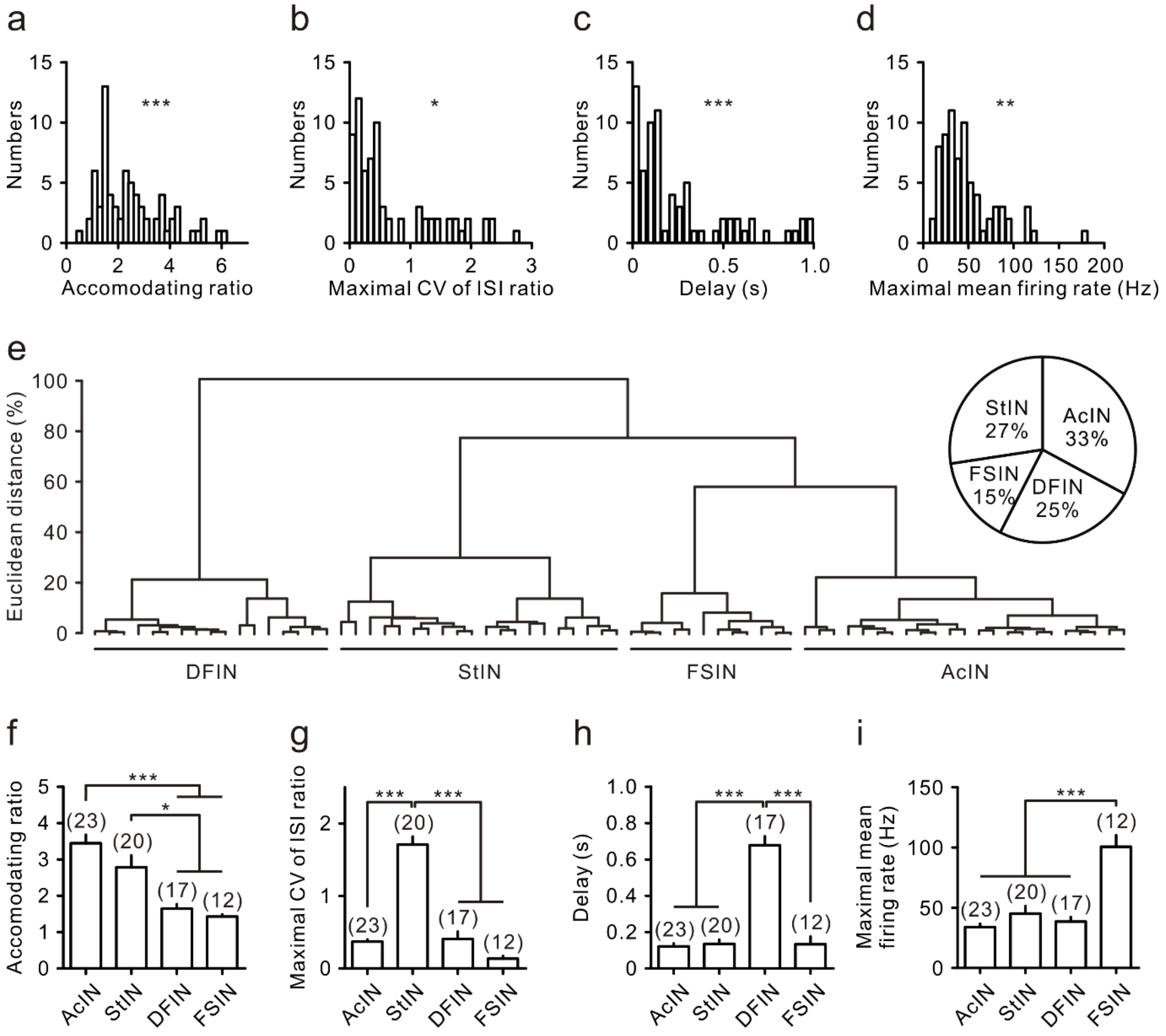

Supplementary Figure S1 | Classification of GABAergic INs in the BLA

(**a-d**), Histograms of accommodating ratios, maximal CVs of inter-spike interval (ISI) ratios, delays of AP and maximal mean firing rates from GFP^+ cells in the BLA. The D'Agostino and Pearson omnibus normality test demonstrates that the properties presented are not unimodally distributed. Asterisks indicate significant deviation from a normal distribution ($^{*}P < 0.05$, $^{**}P < 0.01$, $^{***}P < 0.001$). (**e**) Hierarchical cluster analysis of GABAergic INs performed with electrophysiological

parameters shown in **a**-**d** as the parameters for classification. The x axis of the dendrogram represents the individual cells, and the y axis represents the rescaled distance (squared Euclidean) between groups. Distinct subtypes of INs are marked with bars below the dendrogram. Inset, pie chart shows proportion of each BLA-IN subtype. (**f**-**i**), Bar graphs comparing accommodating ratios, maximal CVs of ISI ratios, delays of AP and maximal firing rates among different IN subtypes ($^{*}P < 0.05$, $^{***}P < 0.001$, Wilcoxon rank-sum test after Kruskal-Wallis test).

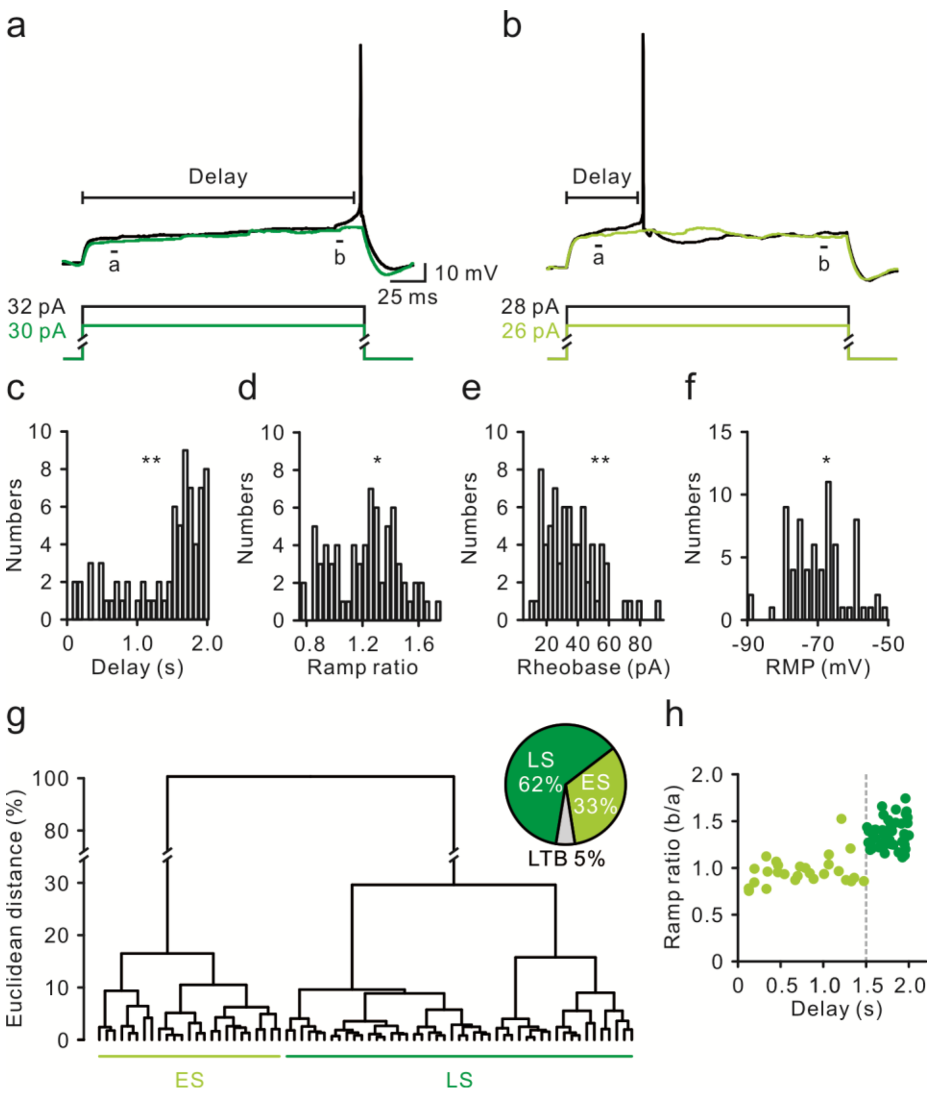

Supplementary Figure S2 | Classification of GABAergic neurons in the CeL

(**a**,**b**), Membrane responses to supra- (black) and sub-threshold (dark and light green) current pulse injections recorded from a representative LS (**a**) and ES cell (**b**). Note the slow depolarizing ramp of the membrane response in the LS cell but not in the ES cell. Bars a and b indicate how the average membrane potentials were measured for the calculation of ramp ratio (b/a). (**c-f**), Histograms of spike delays, ramp ratios, rheobases and resting membrane potentials (RMPs) from non-LTB cells. Asterisks indicate significant deviation from a normal distribution ($^{*}P < 0.05$, $^{**}P$

< 0.01, D’Agostino and Pearson omnibus normality test). (**g**) Dendrogram of non-LTB neurons. The y axis represents the normalized Euclidean distance. Color codes indicate cell classification based on spike delay; cells with spike delay > 1.5 s were dark green otherwise light green. Inset, pie chart shows distinct populations in the CeL. Dark and light green areas denote the major cell populations, LS and ES neurons. Gray area depicts a small subset of LTB neurons. (**h**) Scatter-plot of spike delay vs. ramp ratio. The dotted line indicates the arbitrary cutoff value (spike delay of 1.5 s) that best separates the two populations.

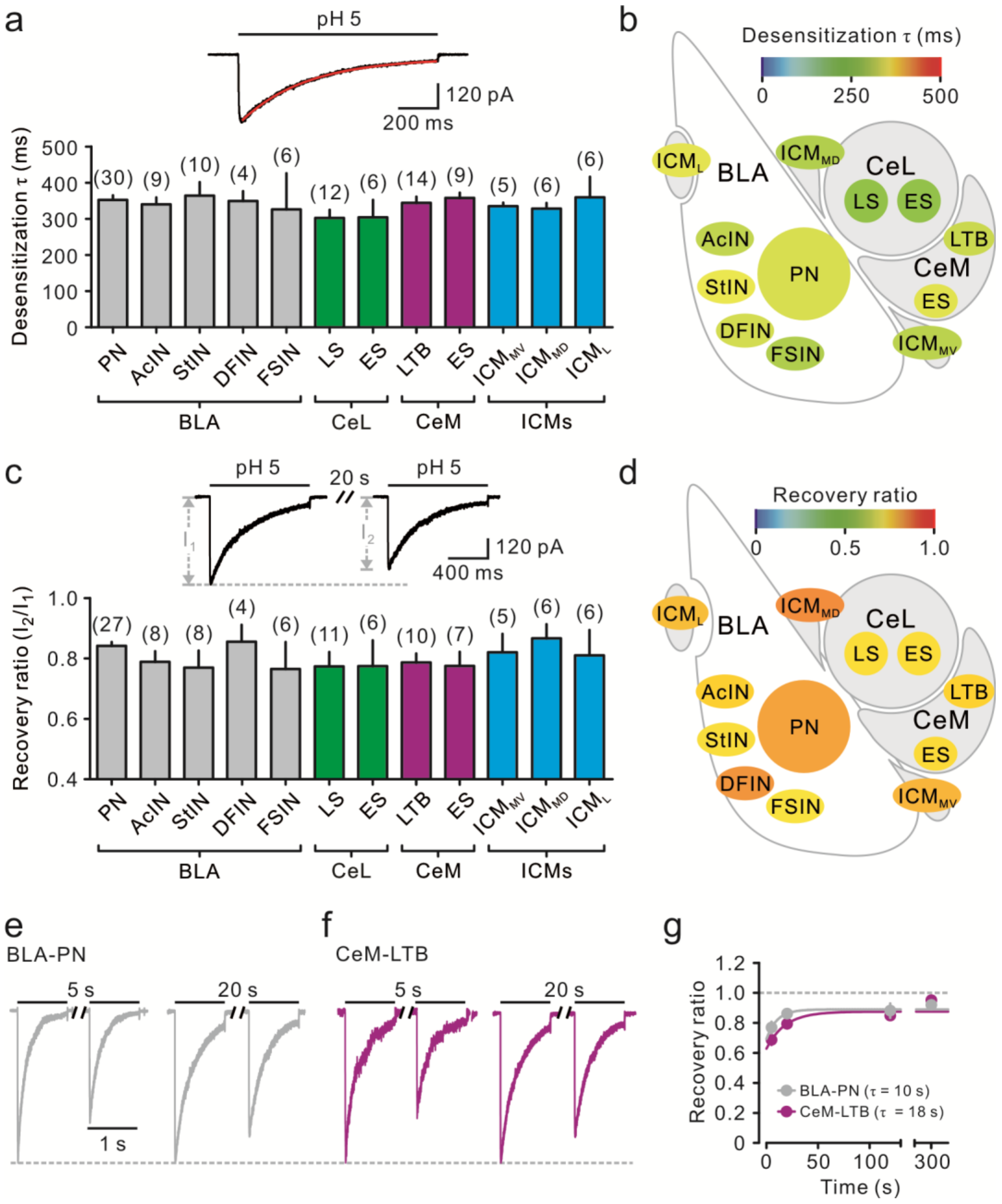

Supplementary Figure S3 | ASIC current kinetics of different amygdala neurons

(**a**) Top, a representative ASIC current trace (black; average of 8 trials) from a BLA-PN; red curve is an exponential function fit. Bottom, bar graph showing desensitization τ of each cell type. P = 0.52, Kruskal-Wallis test. Numbers of patches are given in parentheses above bars. (**b**) Heat map summarizing the desensitization τs of ASIC currents of different amygdala neurons. (**c**) Top, representative traces showing the recovery ratio was measured at -65 mV after rapid changes from

pH 7.4 to 5 for 1 s and then changed back to pH 7.4 for 20 s, followed by a second pulse to pH 5 for 1 s. Bottom, bar graph showing the recovery (I_2/I_1) ratio of ASIC currents of each cell type. P = 0.65, Kruskal-Wallis test. Numbers of patches are given in parentheses above bars. (**d**) Heat map summarizing the recovery ratios of ASIC currents of different amygdala neurons. (**e-f**) Representative traces showing the recovery from desensitization, which were recorded from BLA-PNs (**e**) and CeM-LTB neurons (**f**), was measured with 5 s (left) and 20 s (right) inter-pulse intervals. (**g**) Data points obtained from BLA-PN and CeM-LTB were fitted with a monoexponential function, yielding recovery time constants of 10 s and 18 s, respectively.

Supplemental Tables

Supplementary Table S1 | Electrophysiological properties of neurons in the BLA

	BLA					
	**I PN (23)**	**II AcIN (15)**	**III StIN (11)**	**IV DFIN (10)**	**V FSIN (10)**	**P value**
**RMP (mV)**	-67.1 ± 1.3<br>II, III, IV	-60.1 ± 1.5<br>I, IV, V	-59.6 ± 2.7<br>I, IV, V	-65.5 ± 2.0<br>I, II, III	-68.0 ± 2.4<br>II, III	< 0.001
**R_{in} (MΩ)**	269 ± 40	330 ± 41	298 ± 57	244 ± 28	242 ± 38	0.16
**Sag ratio (%)**	22.9 ± 2.3	25.3 ± 4.3	24.3 ± 3.6	15.1 ± 2.3	22.8 ± 6.2	0.23
**Rheobase (pA)**	72.1 ± 9.7	59.5 ± 11.2	81.9 ± 24.9	91.7 ± 16.0	73.0 ± 19.2	0.45
**Accommodating ratio**	1.89 ± 0.01<br>II, V	3.44 ± 0.23<br>I, IV, V	2.79 ± 0.33<br>IV, V	1.65 ± 0.12<br>II, III	1.43 ± 0.06<br>I, II, III	< 0.001
**Maximal CV of ISI ratios**	0.42 ± 0.07<br>III, V	0.37 ± 0.03<br>III, V	1.71 ± 0.11<br>I, II, IV, V	0.41 ± 0.10<br>III, V	0.13 ± 0.04<br>I, II, III, IV	< 0.001
**Spike delay (ms)**	237 ± 54<br>IV	121 ± 16<br>IV	134 ± 23<br>IV	677 ± 48<br>IV	133 ± 41<br>IV	< 0.001
**Maximal mean firing rate (Hz)**	28.3 ± 1.3<br>II, III, IV, V	33.8 ± 2.9<br>V	45.2 ± 6.2<br>V	38.6 ± 3.7<br>V	100.6 ± 9.6<br>V	<0.001
**AP threshold (mV)**	-37.1 ± 1.4<br>IV	-39.3 ± 2.3<br>IV	-38.4 ± 1.7<br>IV	-34.0 ± 1.5<br>I, II, III, V	-42.3 ± 1.4<br>IV	0.011
**AP half-width (ms)**	1.54 ± 0.11<br>II, III, IV, V	0.82 ± 0.05<br>I, IV	0.78 ± 0.05<br>I, IV	1.18 ± 0.07<br>I, II, III, V	0.64 ± 0.07<br>I, IV	< 0.001
**AP amplitude (mV)**	98.6 ± 1.3<br>II, III, IV, V	91.7 ± 2.5<br>I, III, IV	82.3 ± 2.6<br>I, II, IV	74.6 ± 1.5<br>I, II, III, V	90.6 ± 2.9<br>I, II, IV	< 0.001
**AP maximum rise (V/s)**	320 ± 20<br>IV	311 ± 17<br>IV	270 ± 18<br>IV	210 ± 16<br>I, II, III, V	327 ± 22<br>IV	0.001
**AP maximum decay (V/s)**	-57 ± 15<br>II, III, V	-105 ± 13<br>I, IV, V	-111 ± 10<br>I, IV, V	-59 ± 11<br>II, III, V	-184 ± 8<br>I, II, III, IV	< 0.001

Numbers of cells are given in parentheses. Kruskal-Wallis test was performed to compare the means among groups. All values are given as mean ± s.e.m. Statistical differences between PNs and four types of INs, estimated based on Wilcoxon rank-sum test after Kruskal-Wallis test, are marked under the corresponding parameter, $P < 0.05$. RMP, resting membrane potential; R_{in}, input resistance.

Supplementary Table S2 | Electrophysiological properties of the major cell types in the CeL

	CeL		
	**LS (46)**	**ES (26)**	**P value**
**RMP (mV)**	-70.8 ± 1.4	-65.7 ± 2.2	0.033
**R_{in} (MΩ)**	420 ± 25	415 ± 36	0.038
**Sag ratio (%)**	17.6 ± 2.3	24.3 ± 3.1	0.017
**Rheobase (pA)**	37.6 ± 2.7	31.8 ± 3.0	0.17
**Spike delay (ms)**	1769 ± 24	778 ± 86	< 0.001
**Ramp ratio**	1.37 ± 0.02	0.93 ± 0.02	< 0.001
**AP threshold (mV)**	-34.8 ± 0.7	-33.1 ± 1.1	0.30
**AP half-width (ms)**	2.03 ± 0.11	1.63 ± 0.13	< 0.001
**AP amplitude (mV)**	89.3 ± 1.1	90.5 ± 2.3	0.11
**AP maximum rise (V/s)**	218 ± 9	241 ± 14	0.06
**AP maximum decay (V/s)**	-42 ± 44	-54 ± 25	0.008

Wilcoxon rank-sum test was performed to determine statistical significance between the groups.

Numbers of cells are given in parentheses. All values are given as mean ± s.e.m.

Supplementary Table S3 | Electrophysiological properties of the major cell types in the CeM

	CeM		
	**LTB (37)**	**ES (29)**	**P value**
**RMP (mV)**	-58.0 ± 1.2	-61.7 ± 2.0	0.07
**R_{in} (MΩ)**	568 ± 42	589 ± 50	0.71
**Sag ratio (%)**	43.7 ± 4.0	18.0 ± 1.8	< 0.001
**Rheobase (pA)**	21.1 ± 1.8	33.2 ± 4.0	0.013
**AP threshold (mV)**	-39.5 ± 0.9	-35.8 ± 1.2	0.007
**AP half-width (ms)**	1.17 ± 0.03	1.33 ± 0.07	0.06
**AP amplitude (mV)**	92.5 ± 1.2	91.1 ± 1.6	0.52
**AP maximum rise (V/s)**	254 ± 9	248 ± 10	0.59
**AP maximum decay (V/s)**	-74 ± 38	-66 ± 29	0.05

Wilcoxon rank-sum test was performed to determine the statistical significance between the groups.

Numbers of cells are given in parentheses. All values are given as mean ± s.e.m.

Supplementary Table S4 | Electrophysiological properties of neurons in the ICMs

	ICM_{MV} (16)	ICM_{MD} (12)	ICM_{L} (8)	P value
**RMP (mV)**	-65.3 ± 2.2	-66.3 ± 1.3	-71.1 ± 2.5	0.10
**R_{in} (MΩ)**	693 ± 77	709 ± 60	692 ± 72	0.73
**Sag ratio (%)**	18.1 ± 2.3	18.5 ± 4.6	21.6 ± 5.0	0.54
**Rheobase (pA)**	32.6 ± 3.4	34.9 ± 5.9	37.3 ± 3.3	0.42
**AP threshold (mV)**	-32.9 ± 1.4	-33.5 ± 1.2	-31.8 ± 2.6	0.90
**AP half-width (ms)**	1.14 ± 0.06	1.08 ± 0.04	1.21 ± 0.05	0.25
**AP amplitude (mV)**	86.9 ± 1.9	88.4 ± 1.9	85.6 ± 2.7	0.48
**AP maximum rise (V/s)**	228 ± 12	227 ± 10	200 ± 15	0.28
**AP maximum decay (V/s)**	-72 ± 16	-76 ± 13	-66 ± 9	0.10

Numbers of cells are given in parentheses. Kruskal-Wallis test was performed to compare the means among groups. All values are given as mean ± s.e.m.
